# Cell fusion dynamics: mechanisms of multinucleation in osteoclasts and macrophages

**DOI:** 10.1186/s41232-024-00360-3

**Published:** 2024-11-27

**Authors:** Hideaki Sabe, Yasuhito Yahara, Masaru Ishii

**Affiliations:** 1 Department of Immunology and Cell Biology, Graduate School of Medicine and Frontier Biosciences, Osaka University, Suita, Osaka 565-0871 Japan; 2 WPI-Immunology Frontier Research Center, Osaka University, Suita, Osaka 565-0871 Japan; 3 Department of Orthopaedic Surgery, Graduate School of Medicine, Osaka University, Suita, Osaka 565-0871 Japan

**Keywords:** Cell fusion, Multinucleation, Osteoclasts, Macrophages, Multinucleated giant cells

## Abstract

Cell–cell fusion is a vital biological process where the membranes of two or more cells merge to form a syncytium. This phenomenon is critical in various physiological and pathological contexts, including embryonic development, tissue repair, immune responses, and the progression of several diseases. Osteoclasts, which are cells from the monocyte/macrophage lineage responsible for bone resorption, have enhanced functionality due to cell fusion. Additionally, other multinucleated giant cells (MGCs) also arise from the fusion of monocytes and macrophages, typically during chronic inflammation and reactions to foreign materials such as prostheses or medical devices. Foreign body giant cells (FBGCs) and Langhans giant cells (LGCs) emerge only under pathological conditions and are involved in phagocytosis, antigen presentation, and the secretion of inflammatory mediators. This review provides a comprehensive overview of the mechanisms underlying the formation of multinucleated cells, with a particular emphasis on macrophages and osteoclasts. Elucidating the intracellular structures, signaling cascades, and fusion-mediating proteins involved in cell–cell fusion enhances our understanding of this fundamental biological process and helps identify potential therapeutic targets for disorders mediated by cell fusion.

## Background

Cell–cell fusion is a fundamental biological phenomenon occurring in specific cell types, where the plasma membranes of two or more cells merge, forming a new syncytial cell [1]. This process is essential for various physiological and pathological events, including embryonic development, tissue repair, immune responses, and certain diseases. Examples of physiological cell fusion include the fusion of sperm and oocytes during fertilization [2], the fusion of cytotrophoblast cells into the syncytiotrophoblast for proper placental function [3], and the formation of skeletal muscle cells where mononucleated myoblasts fuse to form multinucleated myotubes [4].

One of the most intriguing examples of physiological cell fusion is the formation of osteoclasts, which absorb bone matrix and contribute to bone remodeling throughout life [5–7]. Osteoclast formation involves the fusion of mononuclear monocyte/macrophage lineage cells in response to specific molecular signals, primarily receptor activator of nuclear factor kappa Β ligand (RANKL) and macrophage colony-stimulating factor (M-CSF) [8–13]. Osteoclasts are highly specialized cells responsible for bone resorption, and their functionality is enhanced by the cell fusion phenomenon. Pathological conditions such as osteoporosis and rheumatoid arthritis (RA) increase the number of activated osteoclasts and nuclei, linking cell fusion to osteoclast function and bone resorption activity [14–16].

In addition to osteoclasts, other multinucleated giant cells (MGCs) differentiate from the monocyte/macrophage lineage [17, 18]. These MGCs are observed primarily during chronic inflammation and foreign body reactions, and their formation process is similar to that of osteoclasts. These cells form by the fusion of monocytes and macrophages in response to stimuli such as inflammatory cytokines (e.g., interferon-gamma (IFN-ɤ), interleukin (IL)-4, IL-13), specific pathogens, and interactions with non-biological materials. Subtypes of MGCs include foreign body giant cells (FBGCs) and Langhans giant cells (LGCs), each seen in specific pathological situations [19–21]. These cells perform various functions, including phagocytosis of large foreign bodies and pathogens, antigen presentation, and secretion of inflammatory mediators [21].

Research on cell–cell fusion, particularly in virology and immunology, has a long history. Enveloped viruses undergo membrane fusion between the viral lipid bilayer and host cell membranes, which is crucial for viral infection [22]. Artificial cell fusion using Sendai virus particles was first reported in 1957 and has since been utilized to study cell biology and genetics [23]. This knowledge led to techniques such as monoclonal antibody manufacture, using cell fusion between B cells and myeloma cell lines [24]. In oncology and infectious diseases, cell fusion can impact cancer progression and pathogen spread, potentially worsening pathological conditions [25, 26]. Despite over 150 years of research since cell fusion was first reported, the molecular processes controlling cell fusion in eukaryotes remain elusive [27].

Given this historical background and the diverse roles of cell–cell fusion in physiological and pathological processes, this review aims to provide a comprehensive overview of cell fusion mechanisms, with a particular focus on macrophage fusion and osteoclast formation. By exploring the molecular intricacies of these processes, we aim to elucidate the underlying mechanisms governing cell fusion in the innate immune system.

## Basic mechanisms of cell fusion

**Fig. 1:**
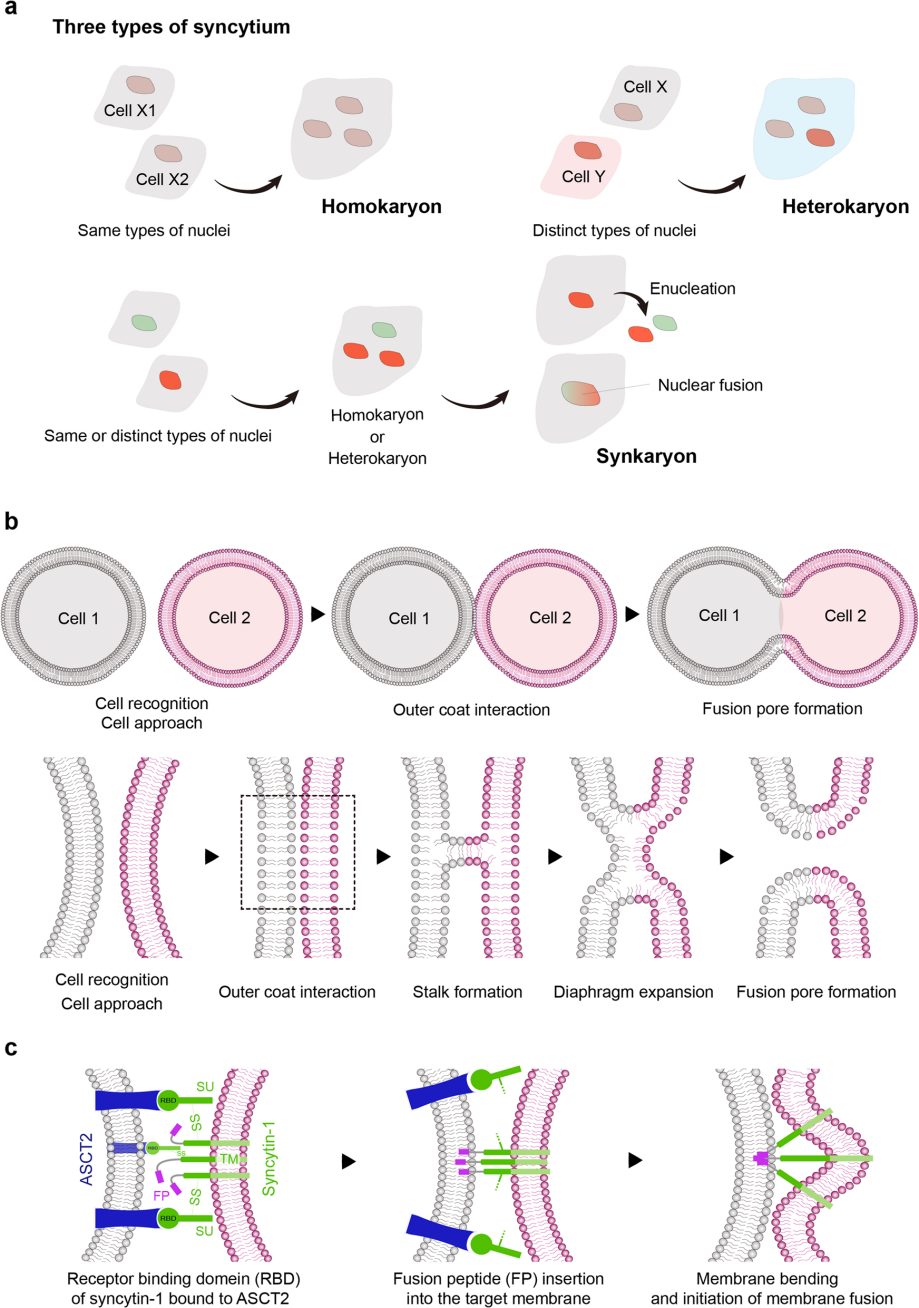
Types of cell fusion and the process of cell membrane fusion. **a **Three types of syncytia: Cells that undergo fusion are collectively referred to as syncytia and include three types—homokaryon, heterokaryon, and synkaryon. Homokaryon formation: Fusion of cells with the same type of nuclei results in a multinucleated cell. Heterokaryon formation: Fusion of cells with distinct types of nuclei forms a multinucleated cell. Synkaryon formation: A single-nucleus cell is formed through the fusion of cells with either the same or different types of nuclei. **b **Membrane fusion process: Cells approach and interact with each other (outer coat interaction). This interaction leads to the formation of a stalk-like structure. The stalk then expands into a diaphragm. Finally, the fusion pore forms, completing the membrane fusion. **c **Syncytin-1 mediated fusion in trophoblasts: The receptor-binding domain (RBD) of syncytin-1 binds to the Na-dependent neutral amino acid transporter type 2 (ASCT2). The surface unit domain (SU) is removed from the transmembrane domain (TM) by cleaving disulfide (SS) bonds. The fusion peptide (FP) is inserted into the target plasma membrane, initiating host plasma membrane bending

Cells resulting from the fusion of somatic cells are generally referred to as syncytia [28–32]. Three distinct categories of syncytia have been defined based on the genetic composition of the fusing cells and their nuclear configuration. Homokaryons arise from the fusion of genetically identical cells, resulting in a multinucleated structure [33, 34]. Examples of homokaryons include those observed in placentation, skeletogenesis, and osteoclast-mediated bone resorption [29, 35]. In contrast, heterokaryons are formed by the fusion of genetically distinct cells, producing a multinucleated entity containing nuclei from diverse origins [36, 37]. This category includes phenomena such as tumor-host cell fusion events, which can confer novel functionalities to the resulting hybrid cells [27, 29, 38, 39]. In addition, synkaryons represent a unique category characterized by a single nucleus formed through the fusion of cells with either identical or different types of nuclei [40, 41]. The presence of a single nucleus after cell fusion requires nuclear fusion and the selective loss of chromosomes while maintaining cell viability. If nuclear fusion does not occur, a synkaryon can result from the shedding of an intact nucleus from a homokaryon or heterokaryon (Fig. 1a). Examples of synkaryon formation include the generation of hybridomas for monoclonal antibody production and tumor cell fusion [41, 42].

Our current understanding of cell fusion mechanisms largely stems from studies of virus-cell fusion processes [22], which have provided valuable insights into the general principles of membrane fusion. Enveloped viruses, which acquire their outer coats from the plasma membrane, enter target cells through a fusion process that merges their viral envelope with the plasma membrane of the host cell [27]. The membrane fusion process encounters several energy barriers that must be overcome for successful cell fusion. One significant component is the hydration force, which arises when opposing membranes come within approximately 10–20 Å of each other [43]. Additionally, the membranes must undergo significant deformation, typically forming a nipple-like protrusion, to initiate fusion. This structural rearrangement requires energy to overcome the membrane’s resistance to bending. The energy required to reach the hemifusion state is estimated to be a few tens of kilocalories per mole [22, 44, 45]. Fusogens, proteins that facilitate membrane fusion, lower this kinetic barrier and catalyze the fusion process. Viral fusion proteins achieve this by using the free energy released during a protein conformational change to pull the membranes together [45]. When the polar heads of phospholipids in the outer coats of two cell membranes approach each other, they form a stalk-like structure that expands to create a hemifusion diaphragm [46, 47]. The tension in the extended membrane then promotes the fusion of the inner coats, forming a fusion pore (Fig. 1b).

Viral fusion is initiated by the binding of a viral fusion protein to a receptor on the target cell [48, 49]. Numerous fusion proteins used by enveloped viruses have been identified [27, 49, 50]. However, identifying eukaryotic fusogens has been more challenging due to the complexity of cell surfaces and the involvement of various biological processes such as differentiation, adhesion, and migration in cell fusion events. Several non-viral fusion proteins have been identified as key mediators of cell fusion events in vertebrates. These proteins belong to diverse molecular families and participate in various physiological processes. For instance, IZUMO1, an immunoglobulin superfamily protein, promotes sperm fusion with oocyte membranes [51]. Meltrin alpha, a member of the disintegrin and metalloproteinase family, is involved in the fusion of myoblasts into skeletal muscle fibers [52, 53]. Additionally, dendritic cell-specific transmembrane protein (DC-STAMP), a seven-transmembrane protein originally identified in dendritic cells or IL-4-stimulated macrophages, mediates macrophage fusion and triggers the differentiation of osteoclasts and MGCs [54–57].

Syncytin-1 plays a crucial role in trophoblast differentiation during placental formation by promoting cell fusion through interactions with the Na-dependent neutral amino acid transporter type 2 (ASCT2) localized on the target cell membrane [30, 58]. Syncytin-1 controls human trophoblast fusion through its retroviral envelope-like properties [31]. Syncytin-1 is a glycoprotein composed of a transmembrane domain (TM) and a surface domain (SU) linked by a disulfide bond. The receptor binding domain (RBD) located in the syncytin-1 SU subunit is recognized by ASCT2, inducing a conformational change [59]. The SU subunit then dissociates from the TM subunit, triggering a loop-to-helix movement of the fusion peptide in the SU subunit. This fusion peptide is then inserted into the neighboring cell’s membrane at a distance of approximately 100 Å [60]. Once the fusion peptides have been embedded into the target membrane, the trimer of TM subunits initiates plasma membrane bending, merging, and fusion-pore formation (Fig. 1c) [29]. Although the mechanisms underlying eukaryotic cell fusion are complex and involve the interaction of multiple molecular processes, elucidating these mechanisms—both physiological and pathological—holds the potential for revolutionary advances in developmental biology and reproductive medicine.

## Origins of monocytes, macrophages, and osteoclasts

Monocytes and macrophages are essential components of the innate immune system, performing critical functions such as phagocytosis and activating the adaptive immune response [61, 62]. Unlike other immune cells, macrophages possess the unique ability to fuse with one another to form MGCs [5, 6]. Recent research has revealed that macrophages have multiple developmental origins: erythromyeloid progenitors (EMPs) in the embryonic yolk sac and hematopoietic stem cells (HSCs) [63–67]. EMPs give rise to a population of tissue-resident macrophages during embryonic development. These EMP-derived macrophages arise independently from the HSC lineage and persist in many tissues throughout adulthood. Similar to macrophages, osteoclasts also share these developmental origins [68, 69]. Traditionally, HSC-derived monocytes/macrophages have been considered the primary source of osteoclast precursors, a concept supported by numerous in vitro studies demonstrating the fusion of monocyte-derived precursors to form multinucleated osteoclasts [70]. However, recent fate-tracking studies have revealed a more complex picture of osteoclastogenesis in vivo. These studies suggest that while osteoclasts can form through the fusion of precursors from the same lineage, there is also the possibility of cell fusion between EMP- and HSC-lineage monocytes/macrophages, resulting in multinucleated osteoclasts [68]. The discovery of potential cell fusion between EMP- and HSC-derived macrophages and osteoclasts adds another layer of complexity to our understanding of osteoclast origins and development [68, 71].

## Characteristics of multinucleated giant cells

**Fig. 2:**
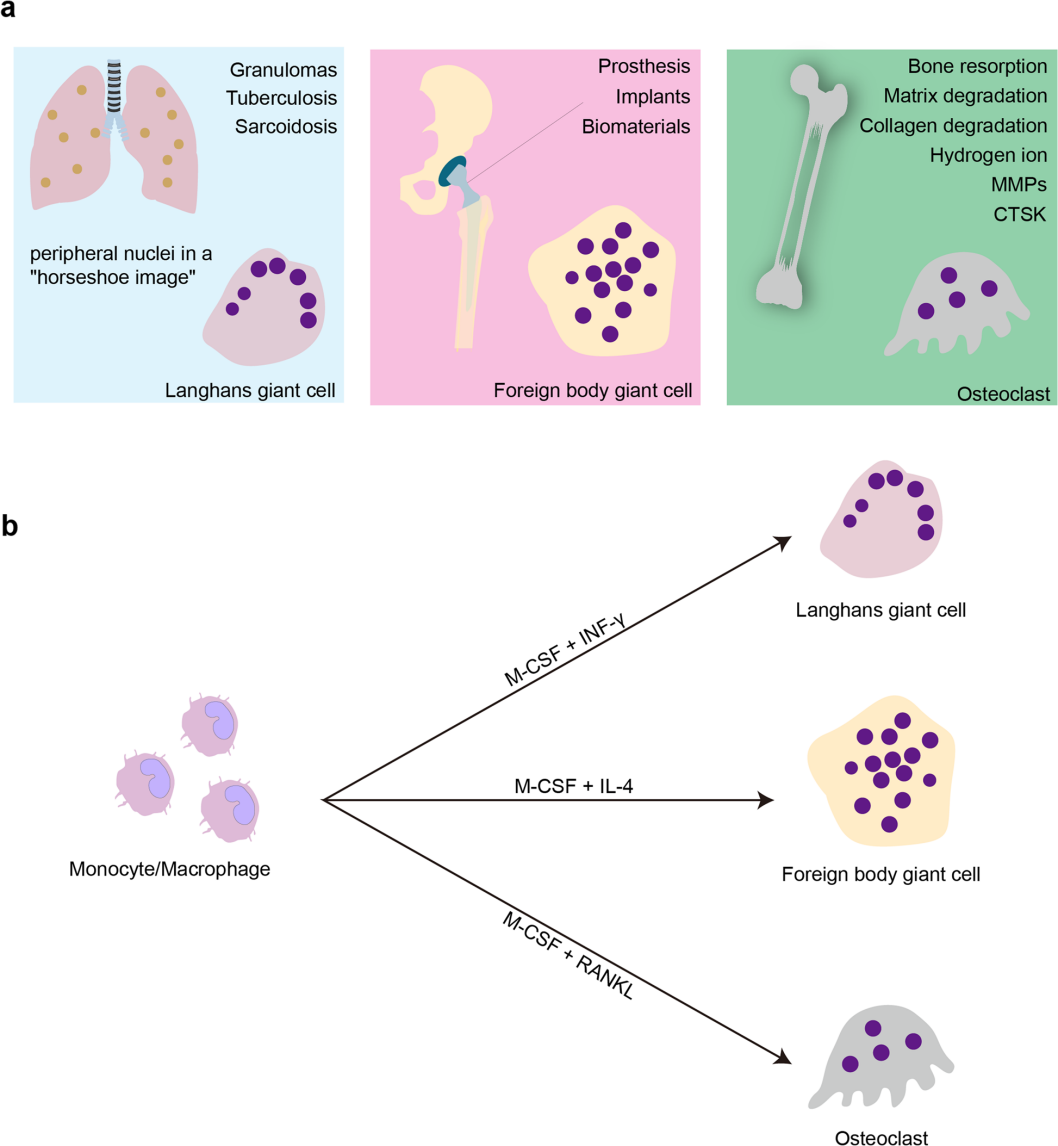
Types and characteristics of multinucleated giant cells. **a **Types of multinucleated giant cells: Langhans giant cells, found in granulomatous conditions such as tuberculosis and sarcoidosis, have nuclei arranged in a horseshoe pattern. Foreign body giant cells, formed in response to foreign materials, have randomly distributed nuclei within the cytoplasm. Osteoclasts, involved in bone resorption, are characterized by their large size and multiple nuclei. **b **In vitro differentiation of multinucleated giant cells: Macrophages can differentiate into different types of giant cells when stimulated with specific cytokines. IFN-γ stimulation leads to the formation of Langhans giant cells. IL-4 stimulation results in the differentiation of macrophages into foreign body giant cells. RANKL induces the formation of osteoclasts, which are involved in bone resorption

Based on their morphology and functional characteristics, MGCs are generally subdivided into three main subtypes: osteoclasts, FBGCs, and LGCs (Fig. 2a) [72]. Osteoclasts, which degrade bone, play a vital role in bone remodeling under physiological conditions [7]. However, they are also involved in the pathogenesis of RA, osteoporosis, and cancer bone metastasis, indicating that proper regulation of osteoclasts is crucial to prevent pathological conditions [73]. Recent studies suggest the existence of disease-related osteoclasts with functions that differ from those observed in healthy states, particularly in patients with RA or cancer. Consequently, these disease-related osteoclasts may contribute to the pathogenesis of these conditions, and specific therapies targeting pathogenic osteoclasts are currently under investigation [74, 75].

In contrast, FBGCs and LGCs appear exclusively under pathological conditions. FBGCs are associated with inflammatory reactions against foreign materials, including implants and prostheses [76, 77]. LGCs are found in granulomas of both infectious and non-infectious diseases [78]. In vitro experiments have shown that macrophages can give rise to osteoclasts, FBGCs, and LGCs when treated with RANKL, IL-4, and IFN-γ, respectively (Fig. 2b) [21, 72, 79]. Each subtype has a distinct nuclear distribution: osteoclast nuclei are centrally located within the syncytium [80], LGCs display a ring of nuclei along the cell border, and FBGCs have scattered nuclei throughout the cytoplasm [21].

**Fig. 3:**
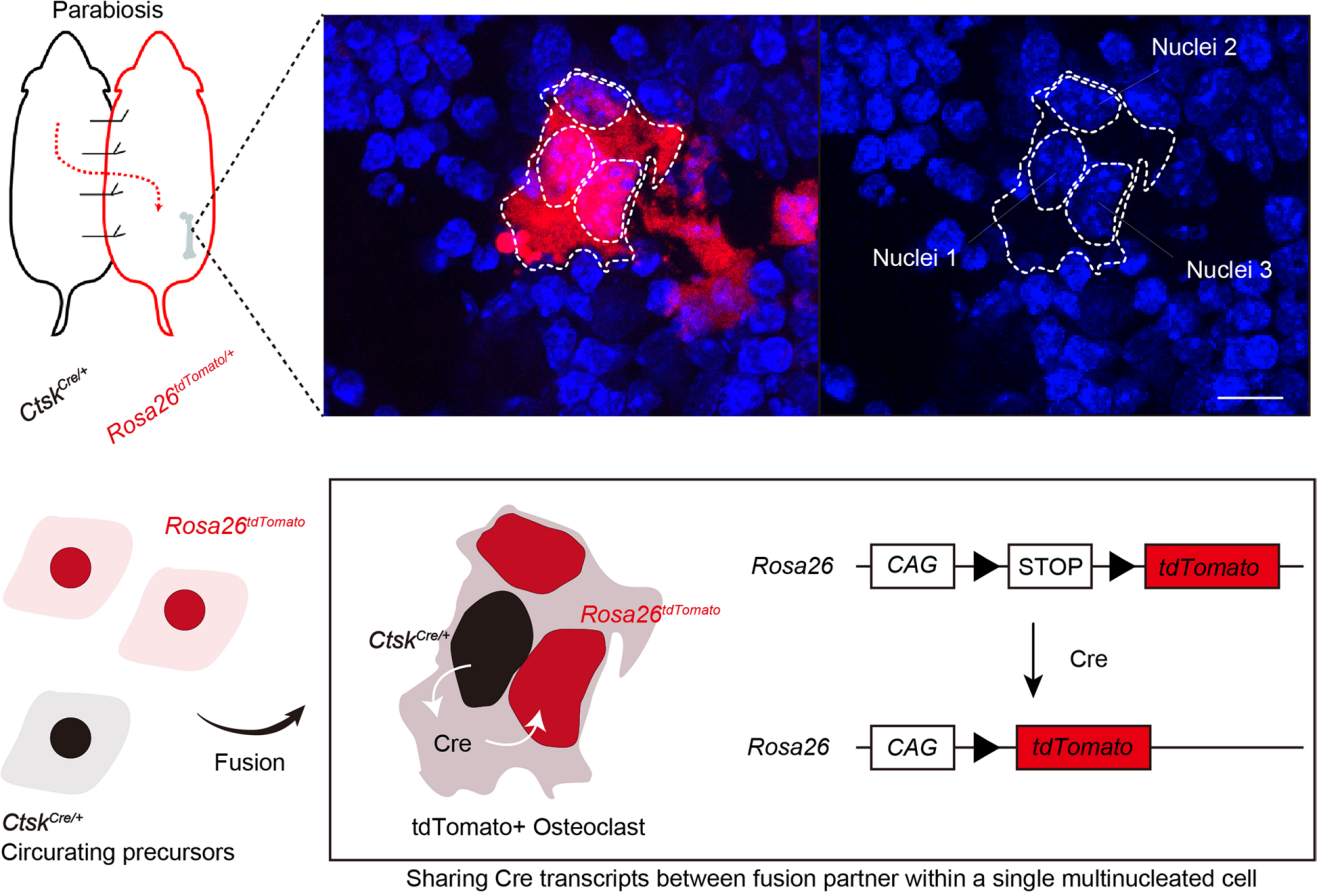
Multinucleated cells share transcription products within a single cell. A parabiosis experiment was conducted with a mouse expressing Cre under the control of the *Ctsk* gene and a tdTomato reporter mouse. When their circulations are shared, cells differentiate into tdTomato-expressing osteoclasts through cell-cell fusion. This occurs because the Cre protein induces recombination between loxP sites, resulting in the elimination of the STOP sequence. This demonstrates that multinucleated cells share transcription products within a single cell. Scale bar, 10μm

The phenomenon of multinucleation raises questions about transcriptional regulatory systems, as transcripts derived from multiple nuclei are shared within a single cell. This can be demonstrated through a simple parabiosis experiment using the Cre-LoxP system. In this experiment, one mouse expresses Cre under the control of the osteoclast marker cathepsin K (CTSK) (*Ctsk*^*Cre*^), whereas the other is a tdTomato reporter (*Rosa26*^*loxp−STOP−loxp−tdTomato*^). When both circulations are shared, the cells differentiate into tdTomato-expressing osteoclasts via cell–cell fusion, as they share the Cre protein and reporter sequence within a single cell (Fig. 3). Although the transcriptional regulatory machinery in multinucleated cells with multiple nuclei remains largely unknown, recent single-nucleus RNA technologies have elucidated nuclear heterogeneity within syncytial skeletal muscle cells [81, 82]. Given that the regulatory system of multinucleated cells is presumed to be more complex than that of mononucleated cells, further investigation is necessary to fully understand transcriptional regulation in MGCs.

## Molecular mechanisms underlying cell–cell fusion

**Fig. 4:**
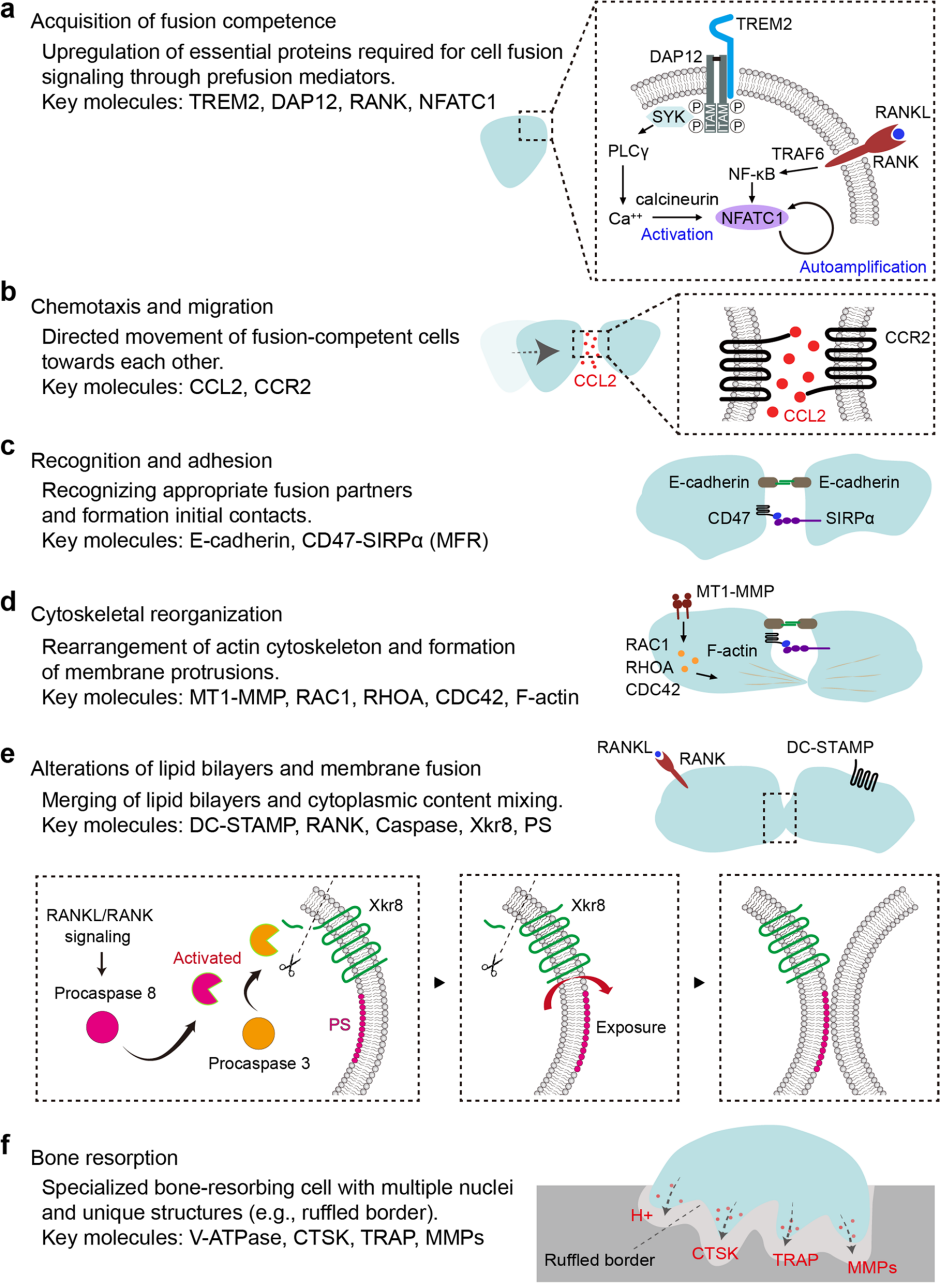
Molecular mechanisms of cell-cell fusion in osteoclastogenesis. **a **Acquisition of fusion competence: Stimulation of TREM2 leads to DAP12 phosphorylation, activating SYK and ZAP70. This triggers pathways that, along with RANKL/RANK signaling, enhance the induction of NFATC1, which is a master regulator of osteoclast differentiation. **b**, **c **Chemotaxis and adhesion: CCL2/CCR2 signaling mediates chemotaxis and migration, facilitating fusion competency. Cell-cell adhesion is mediated by E-cadherins and SIRPα/CD47 interactions, which are crucial for initiating and maintaining the cell contacts necessary for fusion. **d **Cytoskeletal reorganization: GTPases, including RAC1, CDC42, and RHOA, which are regulated by MT1-MMP, orchestrate the dynamic rearrangement of the actin cytoskeleton, resulting in membrane protrusions. **e **Alterations of lipid bilayers and membrane fusion: RANKL/RANK signaling activates caspase-8 and caspase-3, leading to Xkr8-mediated PS exposure. The fusion partner recognizes PS, which is essential for multinucleation. **f **Bone resorption: Osteoclasts secrete H+, CTSK, TRAP, and MMPs from the ruffled border onto the bone surface, dissolving the bone

Cell–cell fusion between macrophages and osteoclasts is hindered by a significant kinetic barrier. Despite this, the rationale for MGC fusion remains largely unclear. Before the actual fusion of MGCs, macrophages undergo several preparatory stages: acquisition of fusion competency, chemotaxis, cell–cell adhesion, and membrane fusion [83, 84] (Fig. 4). Although the stimuli initiating fusion vary among different MGCs, the fusion process is thought to utilize a common molecular machinery among osteoclasts, FBGCs, and LGCs [1].

### Acquisition of fusion competence

The fusion of macrophages or osteoclast precursors is initially achieved through the upregulation of essential proteins required for cell fusion [1, 85]. These key proteins are induced by signaling through prefusion mediators expressed on macrophages [85, 86]. The signaling adaptor DNAX-activating protein 12 (DAP12), a co-stimulatory immunoreceptor tyrosine-based activation motif (ITAM)-bearing factor, is essential for macrophage fusion [87–90]. Additionally, the triggering receptor expressed on myeloid cells 2 (TREM2) is necessary for macrophage multinucleation, mediated by its interaction with the co-stimulatory DAP12 [90]. Following recognition of its unknown ligand, TREM2 associates with DAP12, resulting in the phosphorylation of the ITAM within the cytoplasmic domain of DAP12 [88, 91]. Phosphorylated DAP12 then interacts with cytoplasmic spleen tyrosine kinase (SYK) and zeta chain of T-cell receptor-associated protein kinase (ZAP70), triggering various downstream pathways [91]. Although all MGC subtypes rely on DAP12/TREM2 signaling, distinct downstream pathways contribute to their fusion-competent state [1].

In osteoclast precursors, SYK signaling activates Ca^2^⁺ signaling pathways downstream of phospholipase C (PLC)-γ [88]. This Ca^2^⁺ signaling enhances the induction of the nuclear factor of activated T-cells c1 (NFATC1), the master transcription factor in osteoclastogenesis [86, 88, 92]. In FBGC precursors, IL-4 and ITAM signaling cooperatively activate signal transducer and activator of transcription 6 (STAT6), prompting the expression of essential fusion proteins such as E-cadherin, DC-STAMP, and matrix metalloproteinase 9 (MMP9) [93–96]. In LGC precursors, the downstream signaling pathways remain unclear.

The purinergic receptor P2X, ligand-gated ion channel 7 (P2RX7), an adenosine triphosphate (ATP)-gated ion channel, also plays a role in macrophage multinucleation [97–100]. This receptor detects and transduces signals from extracellular ATP and adenosine, influencing the fusion process. Notably, macrophages expressing high levels of P2RX7 exhibit an abnormally high propensity to form MGCs [101, 102], underscoring the importance of this receptor in the fusion mechanism. In addition, P2X7 blockers have been shown to effectively inhibit macrophage multinucleation [102]. When transiently stimulated with ATP, P2RX7 functions as a cation-selective channel. However, upon repeated ATP stimulation, it transforms into a nonselective pore, allowing hydrophilic molecules up to 900 Da to pass through [98, 103]. This unique property has led to the hypothesis that P2RX7 may drive the fusion process by forming a “fusion pore,” facilitating cytoplasmic connections between neighboring cells [103, 104]. This P2RX7-mediated process has been reported to correlate with Ca^2^⁺ concentration or the activation of caspase-3, which will be discussed later [103]. However, it remains unclear whether this represents one step in a series of fusion phenomena induced by RANKL and IL-4 stimulation or if it is an independent and distinct process.

### Chemotaxis and adhesion

The chemotaxis and migration of macrophages represent the initial phase in cell–cell fusion. C–C motif chemokine ligand 2 (CCL2) and its receptor, C–C motif chemokine receptor 2 (CCR2), are critical mediators of monocyte/macrophage chemotaxis [105–107]. CCL2 not only facilitates chemotaxis but also enhances fusion competency by inducing essential fusion proteins such as DC-STAMP and MMP9 and activating ras-related C3 botulinum toxin substrate 1 (RAC1), a key regulator of the cytoskeleton [108–110]. Following chemotaxis, macrophages undergo cell–cell adhesion, allowing their membranes to come into close proximity before fusion occurs. E-cadherins mediate these homotypic cell–cell contacts, and some studies suggest that integrins also play a role in promoting these interactions and facilitating cell fusion [76, 93, 111]. E-cadherins, which are calcium-dependent adhesion molecules, are crucial for initiating and maintaining cell–cell contacts. They form homophilic interactions between adjacent cells, providing the initial tethering necessary for fusion. Integrins, in contrast, are heterodimeric transmembrane receptors that mediate both cell–matrix and cell–cell adhesion.

The macrophage fusion receptor (MFR), also known as signal regulatory protein alpha (SIRPα), is a transmembrane protein belonging to the immunoglobulin superfamily. Cluster of differentiation (CD) 47 is a ligand for MFR, and the CD47-MFR axis, commonly known as the “don’t eat me” signal, plays an important role in physiological tissue homeostasis [112–114]. MFR and CD47 are also upregulated at the onset of fusion, indicating their involvement in macrophage fusion [112, 115].

### Cytoskeletal reorganization

Cytoskeletal alterations are implicated in multiple facets of macrophage and osteoclast fusion, including chemotaxis, migration, adhesion, the fusion process, and cellular reorganization [85, 116]. Macrophages undergoing fusion exhibit actin-based membrane protrusions, which serve as triggers for the fusion process [84, 117]. Most fusion events occur at these protrusions, and their impaired formation hinders macrophage fusion [84]. Key molecules involved in actin regulation include the Rho-related small guanosine triphosphatases (GTPases), particularly RAC1, cell division cycle 42 (CDC42), and ras homolog family member A (RHOA) [116, 118, 119]. These GTPases orchestrate the dynamic rearrangement of the actin cytoskeleton, which is essential for the formation of membrane protrusions and the execution of the fusion process. Recent studies have also revealed that these GTPases can be regulated by metallothionein 1 (MT1)-MMP [120]. MT1-MMP is a member of the MMPs, a group of endopeptidases known for their ability to degrade various components of the extracellular matrix (ECM). MMPs can modulate macrophage fusion by degrading ECM and cell surface-bound molecules involved in the fusion process [120, 121]. Although MT1-MMP is known to positively regulate macrophage fusion, MMP8 and MMP13 have been reported to have negative effects on this process [120, 122, 123].

### Structural change in lipid bilayers

The plasma membrane of mammalian cells consists of an asymmetric phospholipid bilayer. Typically, aminophospholipids such as phosphatidylserine (PS) and phosphatidylethanolamine (PE) are located in the inner leaflet facing the cytoplasm, whereas phosphatidylcholine and sphingomyelin are predominantly found in the outer leaflet [124]. This asymmetry can be disrupted under various conditions. For instance, PS externalization to the cell surface is a common phenomenon in apoptotic cells, acting as an “eat me” signal that macrophages recognize and phagocytose [125]. This PS exposure is regulated by “flippases,” which move lipids from the outer to the inner lipid bilayer, and “scramblases,” which redistribute lipids in both directions between the bilayers [126].

Recent studies have shown that PS exposure occurs not only in phagocytosed apoptotic cells but also during the fusion process of macrophages [127, 128]. Inhibition of these lipid transporters prevents PS exposure and the subsequent fusion of osteoclast precursors. Additionally, anti-DC-STAMP antibodies have been found to inhibit PS exposure, suggesting that DC-STAMP may play a role in this process during macrophage fusion [127, 128].

In osteoclastogenesis, RANKL-mediated activation of caspase-8 initiates a cascade leading to the activation of caspase-3. Caspase-3 then translocates to the plasma membrane, triggering the activation of the phospholipid scramblase XK-related protein 8 (Xkr8). Xkr8-mediated PS exposure promotes the fusion of osteoclast precursors [129]. Recent studies have identified the nuclear RNA chaperone La protein as a regulator of osteoclast fusion, which is related to PS exposure. During osteoclast fusion, La protein appears at the osteoclast surface, promoting their fusion. This role of La protein is independent of its canonical function and involves direct interactions with Annexin A5. Annexin A5 can anchor La protein to transiently exposed PS on the surface of fusing osteoclasts. The disappearance of La from the cell surface and its relocalization to the nucleus in mature multinucleated osteoclasts serves as an off-switch for their fusion activity [130].

In addition to PS, PE exposure has also been implicated in osteoclastogenesis [131]. During the formation of FBGCs, fusion may rely on the recognition of PS by the lipid receptor CD36. CD36 localizes at cell contact sites, and IL-4-induced cell fusion is impaired in macrophages lacking CD36 [132].

### Final stages of the fusion process

Following the acquisition of fusion competency through chemotaxis, adhesion, and crucial cytoskeletal and lipid modifications, the cell fusion process proceeds to its final phase. In this terminal stage, several pivotal elements play a key role, including DC-STAMP or tetraspanins (CD9, CD81) [108, 133, 134]. DC-STAMP is a seven-transmembrane protein essential for macrophage fusion, which includes the formation of osteoclasts, FBGCs, and LGCs [133, 135–138]. During osteoclastogenesis, DC-STAMP is induced by FOS and NFATC1 signaling [139, 140]. Conversely, the Spi-1 proto-oncogene and nuclear factor kappa B (NF-κB) regulate DC-STAMP expression during FBGC formation, with NF-κB and mitogen-activated protein kinases likely involved in LGC formation [137, 140]. It is hypothesized that DC-STAMP facilitates macrophage fusion in a receptor-ligand manner, yet the specific ligand for DC-STAMP necessary for macrophage fusion remains unidentified [54, 141]. Tetraspanins, which are four-span transmembrane proteins, bind to each other and to various other transmembrane proteins [134, 142]. Their ability to interact with multiple molecules allows them to serve as membrane organizers, facilitating the formation of functional protein clusters within tetraspanin-enriched microdomains, some of which may influence macrophage fusion [143]. However, whether they regulate fusion positively or negatively remains controversial. Despite the identification of many proteins involved in osteoclast fusion, it remains unclear how osteoclasts regulate their fusion and achieve the optimal size necessary to fulfill their biological functions.

## Enzymatic activity of osteoclasts and the necessity of cell fusion

To facilitate bone resorption, osteoclasts exhibit strong attachment to the bone surface via podosomes, which are densely interconnected F-actin structures [144–148]. At the onset of this process, podosomes reorganize into an extensive circular pattern known as the sealing zone [149, 150]. The sealing zone delineates an isolated extracellular compartment called the resorption lacuna, which essentially functions as an extracellular lysosome capable of degrading the bone matrix [95, 149, 150]. Osteoclasts secrete a variety of degradative enzymes and acids into these extracellular lysosomes, including CTSK, MMPs, and tartrate-resistant acid phosphatase (TRAP) [105, 151–153]. Maintaining giant multinucleated osteoclasts through cell fusion may enhance bone resorption efficiency by allowing the formation of larger extracellular lysosomes [154]. Furthermore, the presence of multiple nuclei in a single cell increases the capacity for transcription and translation of enzymes, potentially leading to higher concentrations of degradative enzymes in the resorption lacuna. Multinucleation also provides advantages in terms of energy production for bone resorption, as the increased number of mitochondria in multinucleated osteoclasts can support the high energy demands of enzyme secretion and the maintenance of an acidic environment in the resorption lacuna, a topic that will be discussed later.

Additionally, osteoclasts internalize bone remnants for intracellular degradation [155, 156]. These lysosomal enzymes are generally secreted at the periphery of the ruffled border, whereas the uptake of degradation products occurs at the center [156]. This internalization process is coordinated by clathrin-mediated endocytosis and phagocytosis [156]. Consequently, intracellular vesicles filled with bone remnants, referred to as transcytotic vesicles, are formed [157]. These vesicles are transported via transcytotic trafficking to the functional secretory zone, where their contents are released into the extracellular environment [85]. Bone mineral resorption is facilitated by the vacuolar-type H + -ATPase (V-ATPase) proton pump, a lysosomal protein inserted into the ruffled border during bone resorption [155, 158]. V-ATPase releases hydrogen protons within the resorption lacunae, leading to the degradation of bone minerals [159]. CTSK, a cysteine protease, cleaves collagen type I, the most abundant protein in bone. In activated osteoclasts, CTSK is highly concentrated at the ruffled border and is crucial for degrading bone matrix proteins [151, 152, 160]. Given that MMP1, MMP9, and MT1-MMP are localized at the ruffled border, it is postulated that MMPs are also involved in bone matrix resorption [155, 161, 162]. When CTSK expression is suppressed, further inhibition of MMPs exacerbates the decline in osteoclast resorptive activity in long bones, suggesting that MMPs may partially compensate for the absence of CTSK [163].

TRAP, a non-specific phosphatase highly expressed in osteoclasts, is particularly detected in transcytotic vesicles [153, 164–166]. Within these vesicles, TRAP aids in degrading bone remnants by catalyzing the production of reactive oxygen species, which in turn damage the internalized bone proteins [167, 168].

## Osteoclast cell fusion regulates their lifespan and energy metabolism

The lifespan and fate of osteoclasts remain subjects of ongoing research. Traditionally, osteoclasts were believed to undergo apoptosis after 2–4 weeks [169, 170]. However, some studies suggest that osteoclasts may survive longer by incorporating new nuclei from circulating precursors [69, 171–173]. Additionally, recent research indicates that high-dose RANKL injections can cause osteoclasts to divide into daughter cells called “osteomorphs” [173]. These findings support the hypothesis that osteoclastogenesis can proceed through the elimination of existing osteoclast nuclei and their replacement with new nuclei derived from fused mononuclear cells under specific conditions. However, further research is necessary to understand this process fully.

Osteoclasts exhibit a transcriptomic signature strongly associated with mitochondrial activity, including oxidative phosphorylation, the tricarboxylic acid cycle, and respiratory electron transport [21, 174]. These ATP-generating pathways are crucial for meeting the unique and significant energy demands of osteoclasts’ bone-resorbing function. It has been reported that multinucleation markedly increases mitochondrial activity in osteoclasts, enhancing both spare respiratory capacity and ATP production [21]. This evidence supports the hypothesis that the continuous supply of new nuclei and mitochondria via cell fusion is key to maintaining mitochondrial activity. However, multinucleation did not increase glycolytic activity, and the specific pathway leading to increased ATP production following heightened mitochondrial activity remains unclear [21]. Additionally, it has been reported that impairment of osteoclast multinucleation reduces bone resorption activity, resulting in osteopetrosis [71]. Further research is needed to fully elucidate the precise mechanisms by which multinucleation contributes to energy maintenance in osteoclasts and its impact on their bone-resorbing function.

## Conclusion

This review provides a comprehensive overview of cell fusion mechanisms, particularly focusing on macrophage and osteoclast formation. It highlights the importance of cell fusion by thoroughly examining the formation, structure, and function of osteoclasts and monocytes/macrophages, as well as their critical roles in various physiological processes. The insights presented in this review not only advance our fundamental understanding of these biological processes but also suggest potential avenues for therapeutic interventions in disorders mediated by cell fusion.

## Data Availability

Not applicable.

## Abbreviations

MGCs: Multinucleated giant cells
FBGCs: Foreign body giant cells
LGCs: Langhans giant cells
RANKL: Receptor activator of nuclear factor kappa Β ligand
M-CSF: Macrophage colony-stimulating factor
RA: Rheumatoid arthritis
IFN-ɤ: Interferon-gamma
IL: Interleukin
DC-STAMP: Dendritic cell-specific transmembrane protein
ASCT2: Na-dependent neutral amino acid transporter type 2
TM: Transmembrane domain
SU: Surface domain
RBD: Receptor binding domain
EMP: Erythromyeloid progenitor
HSC: Hematopoietic stem cell
CTSK: Cathepsin K
DAP12: DNAX-activating protein 12
ITAM: Immunoreceptor tyrosine-based activation motif
TREM2: Triggering receptor expressed on myeloid cells 2
SYK: Spleen tyrosine kinase
ZAP70: Zeta chain of T-cell receptor-associated protein kinase 70
PLC: Phospholipase C
NFATC1: Nuclear factor of activated T-cells c1
STAT6: Signal transducer and activator of transcription 6
MMP: Matrix metalloproteinase
P2RX7: Purinergic receptor P2X, ligand-gated ion channel 7
ATP: Adenosine triphosphate
CCL2: C-C motif chemokine ligand 2
CCR2: C-C motif chemokine receptor 2
RAC1: Ras-related C3 botulinum toxin substrate 1
MFR: Macrophage fusion receptor
SIRPα: Signal regulatory protein alpha
CD: Cluster of differentiation
GTPase: Guanosine triphosphatase
CDC42: Cell division cycle 42
RHOA: Ras homolog family member A
MT1: Metallothionein 1
ECM: Extracellular matrix
PS: Phosphatidylserine
PE: Phosphatidylethanolamine
Xkr8: XK-related protein 8
NF-κB: Nuclear factor kappa B
TRAP: Tartrate-resistant acid phosphatase
V-ATPase: Vacuolar-type H + -ATPase
